# A Smart Multi-Plane Detector Design for Ultrafast Electron Beam X-ray Computed Tomography

**DOI:** 10.3390/s20185174

**Published:** 2020-09-10

**Authors:** André Bieberle, Dominic Windisch, Kerolos Iskander, Martina Bieberle, Uwe Hampel

**Affiliations:** 1 Helmholtz-Zentrum Dresden-Rossendorf, Bautzner Landstraße 400, 01328 Dresden, Germany; d.windisch@hzdr.de (D.W.); k.iskander@hzdr.de (K.I.); m.bieberle@hzdr.de (M.B.); u.hampel@hzdr.de (U.H.); 2 Chair of Imaging Techniques in Energy and Process Engineering, Technische Universität Dresden, 01062 Dresden, Germany

**Keywords:** ultrafast computed tomography, 3D tomography, scintillation detectors

## Abstract

In this paper, a smart detector design for novel multi-plane ultrafast electron beam X-ray computed tomography is presented. The concept is based on multi-plane electron beam scanning on a transparent X-ray target and elongated cuboid-shape scintillation detectors for radiation detection over an extended axial scanning range. The optical part of the scintillation detector acts as both an X-ray sensitive scintillator with a fast time response and a light guide. With that, we reduce detector complexity, number of detector elements, overall power consumption, and detector costs. We investigated the performance of this new multi-plane detector design with an evaluation detector setup that is made of cerium doped lutetium yttrium oxyorthosilicate (LYSO:Ce) as scintillation material and an avalanche photodiode (APD) array. Thereby, we assessed two design variants: A monolithic LYSO bar detector and a sandwich detector made of multiple LYSO crystals and glass light-guides. Both types reveal excellent linear detector responses, long-term stabilities, and comparable signal qualities.

## 1. Introduction

X-ray tomography, which is known from medical diagnostics and non-destructive testing, is increasingly employed to study problems of fluid flow and process engineering [1,2,3,4,5]. In such applications, it is of great importance to have a fast tomographic imaging modality available, as multiphase flows in reactors or other apparatuses are typically transient [6]. Fast and high-resolution radiography is known from, e.g., synchrotrons, where the X-ray intensity is high enough to perform cine X-ray imaging [7,8,9]. There are also approaches for fast X-ray radiography and tomography with conventional X-ray tubes. In reference [10], a tomographic system with three powerful X-ray tubes and three X-ray detector arrays operating in parallel is presented. Hori et al. [11] developed a tomographic scanner with 60 small X-ray tubes being fired subsequently to obtain one slice image in 500 µs. Misawa et al. [12] extended the scanner towards 18 X-ray sources. Morton et al. [13] presented a system basing on a similar principle but with special switchable X-ray tubes and a frame rate of up to 50 frames per second.

In 2008, ultrafast electron beam X-ray computed tomography (UFXCT) for multiphase flow studies was introduced [14]. The functional principle is similar to cine CT [15] but much faster. It enables CT scanning with up to 8000 frames per second at a spatial resolution of approximately 1 mm [14,16]. It was successfully applied in the past to analyze, e.g., gas-liquid two-phase flows in technical devices [17,18,19,20,21,22]. The CT scanner is essentially designed for two-dimensional (2D) imaging in two distinct cross-sectional imaging planes. With that, velocity and axial extension of structures passing both planes can be determined [23]. Three-dimensional (3D) imaging would be, of course, of even higher value for many applications.

**Figure 1:**
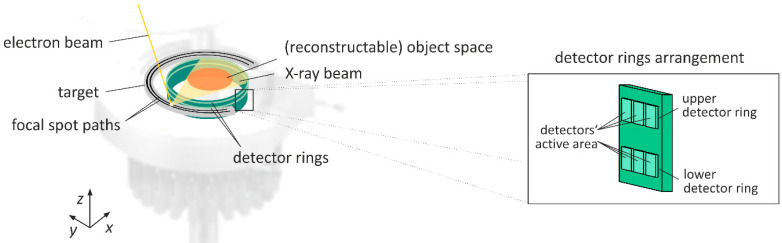
Functional principle of ultrafast electron beam X-ray computed tomography. Dim parts are components with minor functional meaning for computed tomography scans.

**Figure 2:**
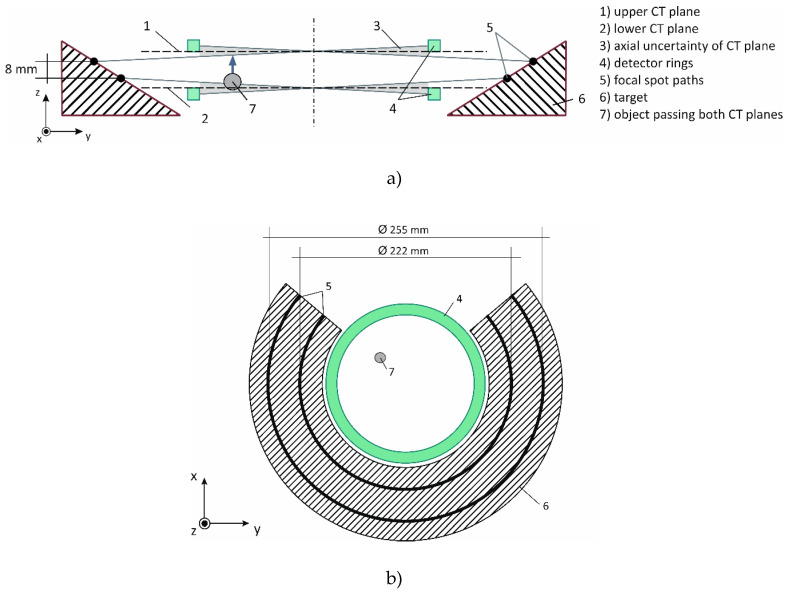
Schematic axial cut view of the ultrafast electron beam X-ray CT arrangement of tungsten target and detector rings: (**a**) Side view (**b**) top view. The focal spot paths usually have a vertical distance of approximately 8 mm. For more details, see [26].

The basic principle of UFXCT is schematically depicted in Figure 1. An electron beam is continuously scanned with up to 8000 revolutions per second across a semi-circular tungsten target and, in this way, creates a rapidly moving X-ray focal spot. Two fixed rings of detector pixels record the X-ray photon flux in current mode. The detector is read-out with a sampling rate of *f*_Samp_ = 1 MHz. The detector pixels are room-temperature semiconductor elements with an active area of approximately 1 mm by 1 mm and an interaction efficiency of approximately 95% for X-ray photon energies of up to 150 keV. Scanning subsequently along two focal spot paths with a slight axial displacement allows what we call dual-plane imaging (see Figure 2). That is, images in two axial positions are generated alternately, which allows us to analyze velocities of structures passing the scanning planes via a cross-correlation technique [23,24,25].

A particularity of this approach is illustrated in Figure 2. As the tungsten target is not transparent for X-rays, both detector rings must be arranged inside the X-ray target. Thus, there is a small axial shift between the X-ray paths and the detector rings that leads to slightly deformed imaging planes as well as to a position-dependent CT plane distance profile that must be considered, e.g., for when recovering velocity information from the images [26]. Additionally, this source-detector arrangement prohibits an upgrade of the technique towards 3D imaging by introducing further detector planes.

In 2011, a new approach for 3D scanning with UFXCT was presented by Stürzel et al. [27]. Here, a specially designed full-circle and transparent X-ray target that allows a sufficient fraction of X-rays to pass through the target was used [28]. Only a single detector ring with multiple pixels is placed behind this transparent target. By steering the electron beam along eight focal spot rings at different target heights, a volumetric scanning is achieved that allows reconstructing a volume of about 20 mm in axial extension. The image reconstruction was performed with a modified Feldkamp-Davis-Kress filtered back-projection algorithm [29]. Stürzel et al. analyzed the imaging quality for different deflection frequencies with up to 500 volumes per second using a phantom.

Despite impressive and successful demonstrations [30], there are some drawbacks to this 3D imaging concept. Firstly, the reconstructable volume section decreases with the target diameter. Secondly, for correct volume reconstruction, a 360° target is required, which is not feasible for many applications. Note from Figure 1 that our scanner head has an opening at the side for pipes and other extended objects, and hence the target has an angle of less than 360°.

Because of the above-mentioned issues, we devise a 3D scanning approach that uses the introduced transparent X-ray target [28] and a new radiation detector concept that is based on a detector design that we originally developed and evaluated for gamma-ray CT applications [31]. As ultrafast electron beam X-ray CT is based on a single electron beam that is continuously deflected onto a tungsten target, X-ray paths on different target heights are addressed successively (see Figure 2). For X-ray detection, one could now foresee commercially available multiple pixel ring detectors, like a number of seamlessly arranged flat panel detectors, as published in reference [8], but this would not be economical. Furthermore, such fixed multiple pixel ring detectors would not allow flexible selection of CT imaging planes via different source paths. Additionally, the time responses of such commercially available and in current mode working detector panels are too slow for UFXCT application where sampling frequencies of more than 1 MHz are required. For these reasons, we propose and evaluate two novel detector designs that use only a single-pixel detector whose scintillation material is elongated over multiple planes—the so-called multi-plane detector (MPD) design.

The paper presents the basic concept of the multi-plane scanning approach (MP-UFXCT) that uses the novel multi-plane detectors. In particular, two multi-plane detector designs with different construction but equal functionality are proposed. To prove and to quantify their functionality, both detector designs are manufactured as eight-channel multi-plane detector modules with respect to their further application at UFXCT scanners. The requirements for the selection of suitable scintillation material, photodetector, and analog electronics are discussed as well as the required signal time responses for UFXCT application. Finally, the achieved signal quality for various detector heights and electron beam currents are evaluated by means of a single detector channel.

## 2. Materials and Methods

### 2.1. Principle of Multi-Plane Detectors for UFXCT Imaging

**Figure 3:**
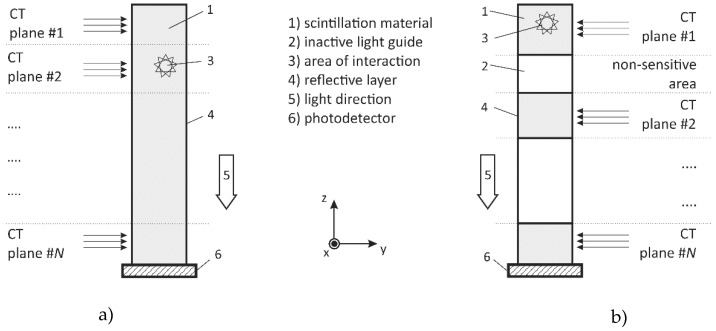
Design of a multi-plane detector applied for multi-plane X-ray computed tomography (UFXCT) applications using (**a**) monolithic scintillation bar or (**b**) sandwich detector (scintillators plus light guides), both coupled to a photodetector.

In Figure 3, the design of two differently composed multi-plane detectors is shown. The scintillator part is of a cuboid shape and extended in length to cover a certain target height. X-rays hitting the detector generate scintillation light, which is guided towards a photodetector at the bottom. All scintillator faces, except the bottom, are coated with a reflective layer. In Figure 3a, a monolithic scintillation bar and in Figure 3b, a sandwich detector design with alternating scintillators and inactive light guides is shown. Though the sandwich design reduces the freedom of CT plane positioning, it precisely defines and collimates the heights of various CT scanning planes, i.e., without any collimator in front of the detector. Furthermore, it is more economical as scintillation materials are typically more expensive than inactive transparent material, such as boron silicate glass. Note, as semiconductor detectors of such lengths, i.e., with a space charge region larger than 2 mm, are prohibitively expensive or cannot be manufactured at all today, such a concept is not considered.

The following scientific problems are addressed for both multi-plane detector designs and were analyzed in this work:

(a) Which scintillation material and photodetector can be applied to obtain sufficient scintillation light yield, time response, and electrical current, respectively, i.e., a sufficient signal-to-noise ratio?
(b) Is a measurable electrical detector current linearly proportional to the X-ray flux at each height of the scintillator and over a wide dynamic signal range?
(c) Is there a quantifiable scintillation light yield reduction with the distance to the photodetector?
(d) What is the influence of additional optically clear material that is stacked between the scintillation materials to enhance the functionality and geometric assembling of the detector design?
(e) What is the cross-talk between individually defined neighboring detector volumes, i.e., how deep interacts X-ray photons outside a masked detector volume height?

For multi-plane UFXCT applications, the following requirements must additionally be considered: (a) A detector time response of better than 1 µs and (b) a modular and stackable multi-plane detector module design.

**Figure 4:**
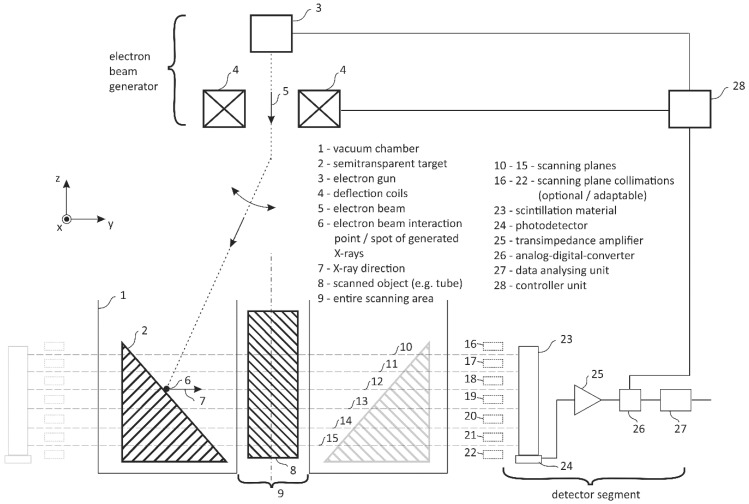
Principle sketch of a prospective multi-plane UFXCT using a certain number of circularly stackable single scintillation detectors extended over a certain CT scanning height.

The principle sketch of a prospective multi-plane UFXCT imaging system is exemplarily shown in Figure 4 for a single multi-plane detector. A sufficiently large number of MPDs must finally be seamlessly arranged circularly behind the transparent X-ray target and must be simultaneously sampled in synchronization with the deflection of the electron beam. Properly dimensioned and spatially adaptable collimators in front of the MPDs can optionally be used to suppress scattered radiation and to set limits to the CT scanning planes.

### 2.2. Experimental Setup

#### 2.2.1. Detector Module

**Figure 5:**
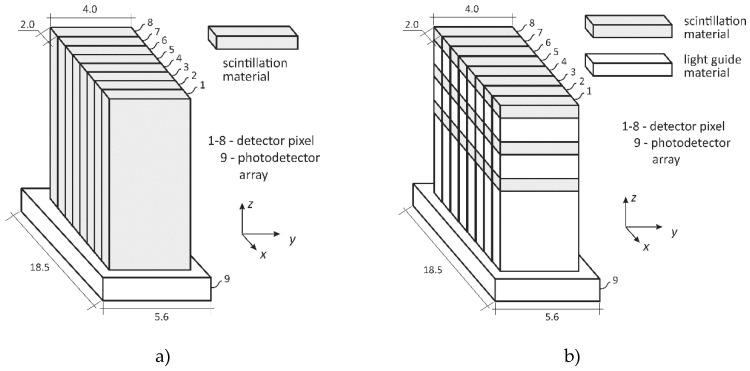
Design of a scintillation (**a**) bar and (**b**) sandwich multi-plane detector (MPD) module with eight detector channels that are seamlessly arranged and coupled to a line array of avalanche photodiodes.

From an earlier eight-channel detector concept [31] and the basic designs in Figure 3 and Figure 4, a suitable design for a scintillation bar and sandwich detector module is proposed in Figure 5. Based on both seamlessly stackable MPD designs, the scintillation material, the photodetector, and a corresponding trans-impedance amplifier circuit are now selected to be able to sample the X-ray photon flux with a frequency of at least 1 MHz.

Firstly, a suitable scintillation material was selected. The decay time of the scintillation process has to be three times faster than the detector sampling frequency (*f*_Samp_ = 1 MHz) to achieve a scintillation signal response of approximately 90% and thus to prevent signal undersampling. In Table 1, the properties of selected optical clear and commercially available scintillation material candidates are listed [32]. Though CsI (TI) offers the best light yield and thus the best signal-to-noise ratio, it has a rather slow decay time of 1 µs as well as an after-glow and thermo-luminescence [33,34]. In addition, BGO was deselected because of the low photon light yield. Eventually, LYSO was chosen because of its characteristics and for economic reasons compared to LSO. However, since LYSO may also suffer from radiation damage effects, the measuring repeatability for UFXCT applications must be experimentally investigated in long-term stability tests because the latest radiation damage knowledge is based on continuous exposure only [35]. Note, long-term means several seconds in this case. Fortunately, the LYSO material also offers the highest density, which meant the best interaction efficiency for X-ray photons and thus the highest possible scintillation light yield.

To achieve a high light collection, i.e., the best signal-to-noise ratio, all faces of the scintillator have to be polished and covered with BaSO_4_ with a layer thickness of 0.1 mm, except the photodetector coupling face. As light guide material, optical borosilicate K9 (BK7) was selected because it is cheap, and it can easily be polished like any other glass. Additionally, its refraction index varies from about 1.53 to 1.5 through the transmission range from 400 to 1400 nm, which is close to the refraction index of LYSO at 420 nm of 1.81. Eventually, optical glue EPO-TEK 301 (Epoxy Technology) was used for connecting the pieces, as it has a spectral transmission of a minimum of 99% in the transmission range of 382 to 980 nm.

Secondly, the photodetector device was selected. Originally, one row of an avalanche photodiode (APD) array S8550 from Hamamatsu was used for single-photon counting [31]. As these APDs are operated in linear amplification mode, the delivered electrical charge is directly proportional to the energy deposited in the scintillator. Further possible photodetector candidates were photo multiplier tubes (PMT), P-I-N photodiodes (PIN-PD), and silicon photo multipliers (SiPM) as all devices offer a sufficient time response for UFXCT applications. PMTs were deselected since they are sensitive to electromagnetic fields, which are permanently present at UFXCT scanners. SiPMs are a promising alternative to APDs. They consist of a large number of APD elements per area that are operated in the so-called Geiger mode. This means electrons generated at each APD element by interacting optical photons are strongly multiplied. This signal amplification leads to an outstanding signal-to-noise ratio that is comparable to classic PMTs. However, while this operating mode is perfectly suited for pulse mode applications, it has some drawbacks for current mode applications. Each APD element needs a so-called recovery time of several nanoseconds in which the photo element has a reduced sensitivity to further optical photons. Furthermore, if the number of incident optical photons per recovery time interval exceeds the number of APD elements, the sensitivity of the SiPM is reduced again. The result is a decreased dynamic analog operating range that limits the suitability of SiPMs for UFXCT applications [36]. The usage of PIN-PDs is generally possible but results in the worst signal-to-noise ratio among all photodetector types. Thus, we decided to use the already successfully applied APD array S8550 [37] for the evaluation MPD module. As already specified in reference [31], two nearby APD elements were electrically connected to obtain an APD line array of eight large detector elements, each with an active area of 1.6 mm by 3.2 mm, a pitch of 0.7 mm, and a temporal response of 1.5 ns.

**Figure 6:**
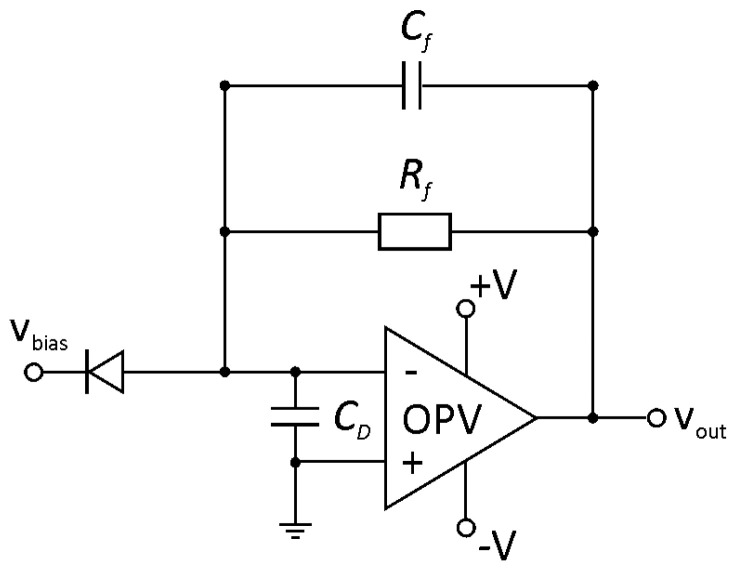
The basic structure of a current-to-voltage trans-impedance amplifier (TIA) for the connection of a biased photodiode.

Eventually, an amplifier stage that is fast enough to follow a dynamic X-ray photon flux within the detector sampling time of 1/*f*_Samp_ was designed. The structure of a common current-to-voltage trans-impedance amplifier (TIA) stage for biased photodiodes is shown in Figure 6. Its cut-off frequency $f_{-3\mathrm{dB}}$ is determined with the feedback resistor

(1) $$R_{f}=\frac{GBP}{2\cdot \pi \cdot C_{D}\cdot f_{-3\mathrm{dB}}^{2}}$$

using the gain-bandwidth product (*GBP*) of the operational amplifier and its corresponding input capacitances $C_{D}$ including the photodetector. The optimal feedback capacitance *C_f_* is calculated by

(2) $$C_{f}=\sqrt{\frac{C_{D}}{\pi \cdot R_{f}\cdot GBP}}$$

#### 2.2.2. Test Setup and Procedure

**Figure 7:**
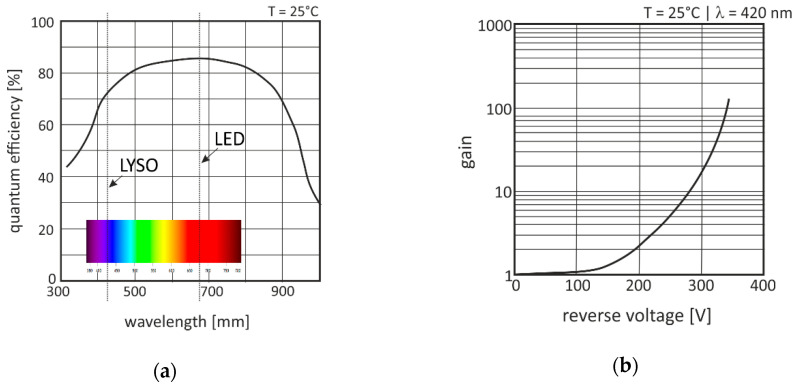
Selected properties of the avalanche photodiode (APD) array S8550: (**a**) Quantum efficiency vs. wavelength and (**b**) internal gain vs. reverse voltage [37].

We designed a test printed circuit board (PCB) for the aforementioned eight-channel MPD composition with a regulated power supply of ±2.5 V. In a first step, the timing performance of the APD elements was validated in combination with the TIA circuit but without scintillation material (LYSO) and X-ray irradiation to isolate their influences. The APDs were exposed for 2 µs with a frequency of 1 kHz by a fast light-emitting diode (LED) with a cut-off frequency of 5 MHz. The emission spectrum peak of the LED (λ = 660 nm) was in the same quantum efficiency range as the LYSO material (see Figure 7a). All APD elements were supplied with a linearly regulated reverse voltage of approximately +300 V at their cathodes that resulted in an internal gain of approximately 20 (see Figure 7b).

**Figure 8:**
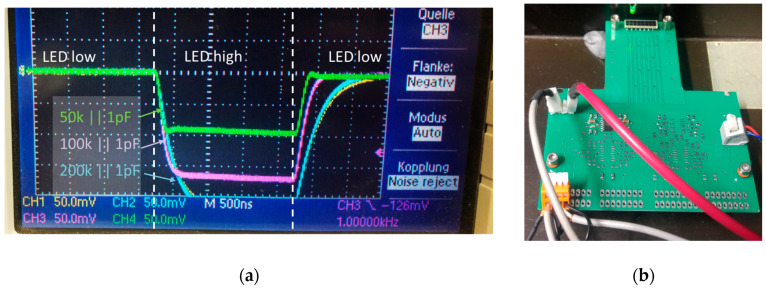
(**a**) Signal response of the differently dimensioned trans-impedance amplifier (TIA) stages coupled to a single APD element (S8550) that is exposed for 2 µs with a fast light-emitting diode (LED) with (**b**) corresponding test setup.

Since precise capacitance values below 1 pF were required (see eq. (2)) and since such values are practically not feasible for surface mounted devices (SMDs), different amplifier feedback pairs were tested in advance (see Figure 8). As a result, a feedback pair of *R*_f_||*C*_f_ = 200 k||1 pF was found to be a good compromise between signal amplification and sufficient time response (*t*_rise_ = *t*_fall_ = 500 ns) that was required for proving the MPD design. The signal offset and the signal root mean square fluctuation was determined to be 3 mV and 0.2 mV, respectively, and was mainly caused by the dark current of each avalanche photodiode, i.e., 300 pA per element [37], and the electronic noise of the amplifier stage.

**Figure 9:**
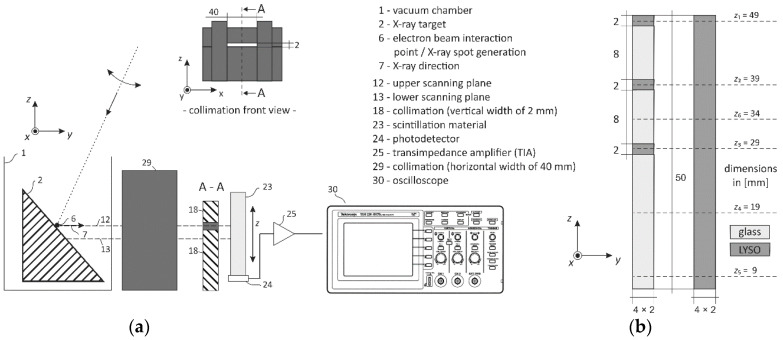
(**a**) Setup to investigate the principle of multi-plane scintillation detectors using (**b**) sandwich (left) and monolithic bar (right) detector design.

Finally, the prepared APD+TIA detector electronics were equipped with scintillator blocks, as shown in Figure 5. To provide the best optical passage between LYSO and APD elements, optical coupling gel BC-330 (Bicron) was glued in between. The final geometry of each scintillation block is shown in Figure 9b. We realized both a monolithic bar block with an LYSO volume per detector pixel of 2 × 4 × 50 mm^3^ and a sandwich block comprising three small LYSO volumes of 2 × 4 × 2 mm^3^ that were connected to light-guiding material of total volume of 2 × 4 × 44 mm^3^. The total detector length, i.e., the maximal scanning height, was chosen as 50 mm for test purposes. However, smaller and even longer scintillator lengths can be fabricated. A single detector channel in the middle of the assembled MPD evaluation module was investigated using the UFXCT scanner, as sketched in Figure 9a. To avoid measuring inaccuracies caused by the electron beam deflection electronics and scattered X-rays, a constant X-ray focal spot path geometry was chosen for all measurements. Instead, the MPD modules were moved along the *z*-axis to realize different exposure heights on the detector. An arrangement of vertical and horizontal collimation was placed in front of the MPD modules (see detail in Figure 9a) such that a radiation passage window of 40 mm (*x,y*-plane) width and 2 mm height (*z*-axis) was left for X-ray exposures.

**Figure 10:**
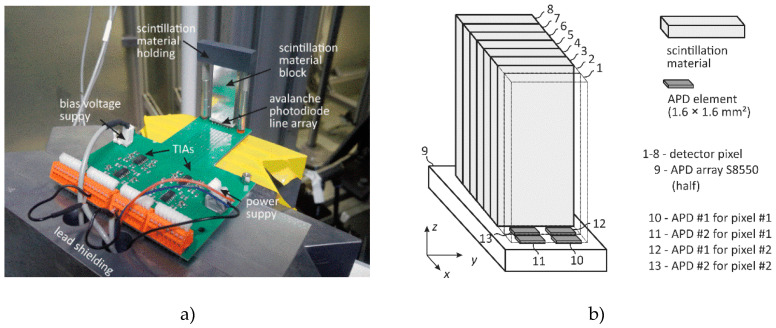
(**a**) Test configuration of a multi-plane detector module and (**b**) details on the coupling of the scintillation blocks onto the corresponding avalanche photodiodes of the APD array (S8550).

The UFXCT scanner was operated with a constant imaging rate of 2000 frames per second, with an acceleration voltage of +150 kV and in dual-plane CT scanning mode. The X-ray spot achieved a velocity of approximately 1.6 mm/µs on the target. As illustrated in Figure 9a, a multi-plane detector exposure interval of about 45 µs per two deflection periods (dual CT scan mode) was provided by proper collimation. The distance between the upper and the lower electron beam path, i.e., scanning planes, was about 8 mm (see Figure 2 and [26]). The analog output voltage signal of the selected inner detector channel (detector pixel #4, see Figure 10b) was sampled with *f*_Samp_ = 2 MHz and a resolution of 24 bit (±5 V) using a commercial data acquisition system (LTT24, Labortechnik Tasler GmbH). Furthermore, the applied APD reverse voltage and the three deflection coil signals (*x-*, *y-* deflection plus electron beam focus) were recorded simultaneously to monitor their correct operation. Eventually, the acquired detector signals were analyzed for various electron beam currents, various detector heights (Figure 9a), and both MPD geometries using GNU OCTAVE v.4.2.2. In Figure 10a, the final MPD test module is shown without collimator, and in Figure 10b, geometric details on the coupling between scintillation blocks and APD array is sketched.

In total, 17 and 12 X-ray exposure measurements with the LYSO bar and sandwich MPD module were performed, respectively. The procedure was always identical: Initially, the MPD module was vertically adjusted to the corresponding X-ray exposure position heights *z*_1_ − *z*_6_. Then, the data acquisition was started without X-ray exposure for approximately 10 s to determine the voltage offset (mean value) and detector noise (deviation), which provided information about the dark current of the detectors. Afterward, the X-ray source was switched on with a constant electron beam current (8, 16, 24, 32, or 40 mA) for approximately 10 s while the X-ray control unit regulated the electron beam flux via its Wehnelt voltage. For long-term stability measurements, the X-ray exposure interval was extended to 30 s. In the end, the X-ray source was switched off, and the detector signal was sampled for another 10 s to determine the dark current of the detectors again. As defined before, both multi-plane detector designs were respectively evaluated with respect to three main topics:

(a) Signal quality, timing and noise
(b) Signal long-term stability
(c) Signal response versus various electron beam currents and detector exposition height

**Figure 11:**
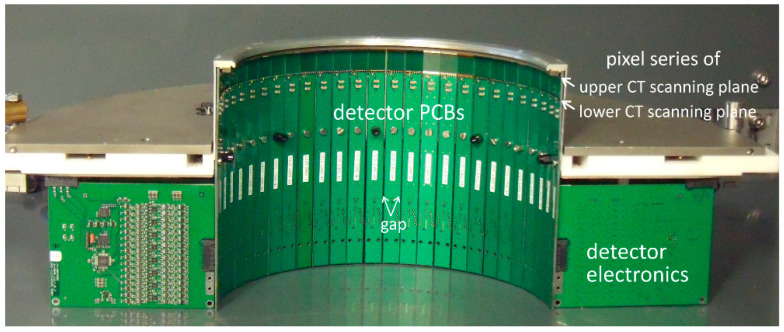
Photography of half of the latest UFXCT detector electronics.

Note, for the studies reported here, both original detector rings of the UFXCT scanner, comprising 18 circular equally distributed detector PCBs, have not been de-assembled. Thus, its structure (see Figure 11) was still between the X-ray source and the MPD module and could additionally be used to analyze the MPDs ability qualitatively to identify sharp object structures, namely the 1 mm gap between each detector PCB. Furthermore, another characteristic was to be expected in the detector signal, namely the structure of the X-ray target that was assembled with a number of discrete brackets whose transition zones induce short X-ray flux reductions [16].

## 3. Results and Discussion

### 3.1. Detector Signal Analysis

**Figure 12:**
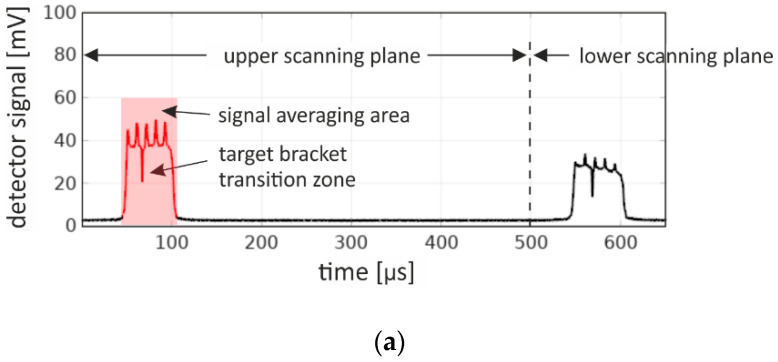

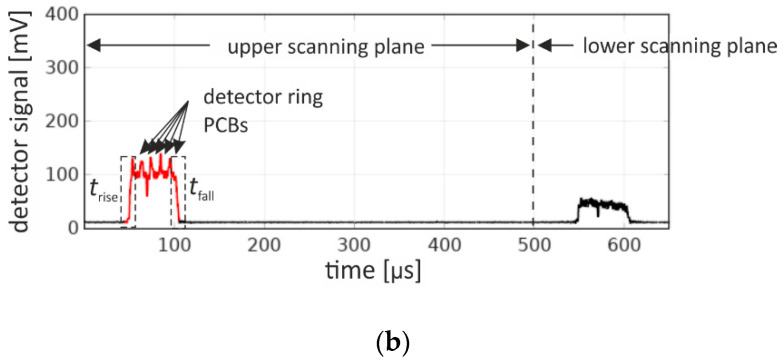
Detector output signals at X-ray exposure height *z*_1_ = 49 mm and for an electron beam current of 8 mA for (**a**) a sandwich and (**b**) a monolithic bar detector. Signals are deflection-synchronized and averaged over 5 s measuring interval. The red marked signal section assigns the region for signal averaging for further investigations at different X-ray exposure heights and electron beam currents to suppress both X-ray statistics and X-ray flux fluctuations.

In Figure 12, the detector output signals of a sandwich (Figure 12a) and a monolithic bar (Figure 12b) multi-plane detector are exemplarily shown for the smallest measuring signal that was expected at an electron beam current of 8 mA and at exposure position *z*_1_ = 49 mm. Both aforementioned structures, i.e., four complete detector PCBs plus their 1 mm gaps and a single transition zone between the two target brackets, could easily be identified in the deflection-synchronized and time-averaged detector signal data of both MPDs. However, despite correct geometric collimation to the upper scanning plane (see Figure 9a), the detector signals clearly revealed an exposure signal in the lower scanning plane. The reasons were the unavoidable parallax effect and the fact that the X-rays emitted from the source did not form a perfectly flat fan-beam but had an angular distribution also in the vertical direction. A signal after-glow caused by the LYSO scintillation material was not verifiable.

**Figure 13:**
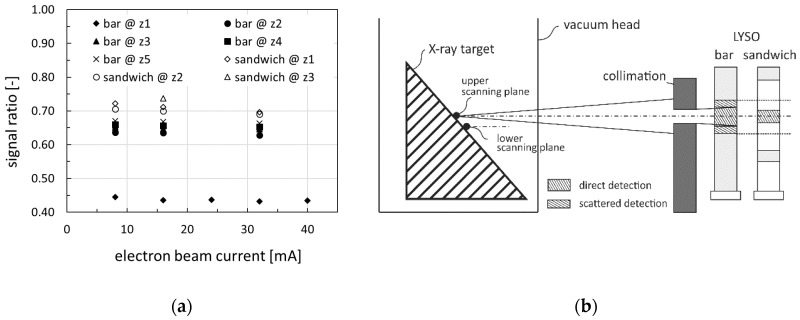
(**a**) Signal ratio between indirectly and directly exposed detectors for different electron beam currents, detector exposure heights, and MPD design configurations. (**b**) Sketch to illustrate the parallax effect, as well as internally measured X-ray scattered radiation.

For a more detailed analysis, the signal ratios between indirectly exposed (lower scanning plane) and directly exposed (upper scanning plane) detectors were calculated for all measurements. The results are shown in Figure 13a. It can be seen that the bar detector signal ratio values were always somewhat smaller than the sandwich detector signal ratios. The reason was the additional scintillation light that was generated by internally scattered X-rays above and below the directly irradiated LYSO bar volume (see Figure 13b). This effect occurred only in the monolithic LYSO bar and hence more at direct exposure than indirect exposure where the amount of non-scattered X-ray photons was lower. Eventually, all the signal ratios obtained from the bar detector at height *z*_1_ significantly differed from the remaining ones. Here, the absent LYSO volume above the detector bar lead to a superimposition of both the missing parallax effect and internal scattering detection. Consequently, only signals obtained from directly exposed detectors were used for further MPD design concept investigations.

As determined from Figure 12, despite the signal strength of the sandwich detector being less than half of that of the bar detector signal, the UFXCT detector ring PCB structure was identified sharper. The reason is to be found in the LYSO material. From the baseline of both signals, i.e., from the non-irradiated detector signal, the mean offset value was determined to be 2.8 mV (sandwich detector) and 11.1 mV (bar detector), respectively. Furthermore, the signal root mean square fluctuation was extracted to be 2.2 mV (sandwich detector) and 5.9 mV (bar detector). As both multi-plane detector designs differed only in its LYSO volume size by a factor of 8.33, this effect was explained by the self-activity of the LYSO material [38]. That is, an additional amount of scintillation light pulses was continuously generated inside the scintillation material by the naturally occurring radioactive isotope ^176^Lu. Its time-averaged offset value can be subtracted from the measured signal, but its fluctuation still influenced the quality of the measured signal, especially at low radiation fluxes. However, in investigations at higher electron beam currents, the observed reduction of object reproduction was found to be negligible. Finally, the time response (rising and falling time) at the MPDs was determined to be 5 µs. The slower time response, compared to the optically determined 500 ns, was explained by (a) the X-ray cone-beam geometry, (b) the parallax effect, generated by the 40 mm horizontal collimation width, and (c) the finite velocity of the X-ray spot. However, results showed that there was no performance difference between bar and sandwich MPD module.

### 3.2. Long-Term Signal Stability

**Figure 14:**
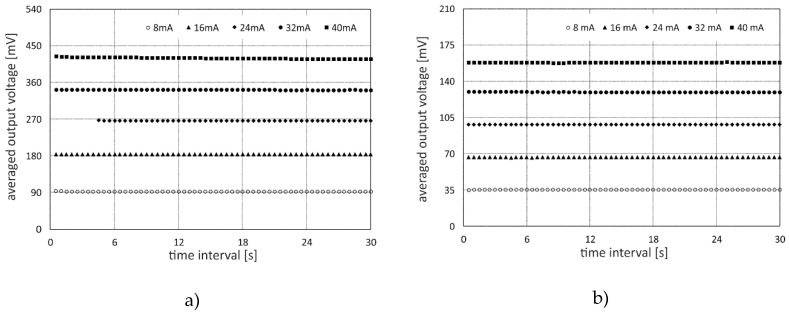
Long-term signal stability analysis of a multi-plane detector assembled with a lutetium yttrium oxyorthosilicate (LYSO) (**a**) bar and (**b**) sandwich block and exposed at various electron beam currents and at constant detector height of *z*_1_ = 49 mm.

For long-term stability investigations, detector signals with an X-ray exposition time interval of 30 s at scanning height *z*_1_ = 49 mm and various electron beam currents were analyzed for both MPD modules. The signal data were divided into 60 segments of 0.5 s that were again deflection-synchronized and time-averaged to reduce statistical errors from signal investigations. Finally, the direct exposure interval (see Figure 12, red-marked signal) was averaged. In Figure 14, the obtained temporal sequences for the bar and sandwich detector is shown for different electron beam currents. As can be seen, there was no signal drifts recognizable for any MPD configuration and operating condition except for 40 mA electron beam current applied to the bar detector module. Here, the end value differed approximately 1.5% from the start value. As this configuration applied the highest X-ray dose into the LYSO material, radiation damage seemed to begin to affect the measurement. In the end, the residual measurements with 10 s X-ray exposure time revealed no signal shifts over time. Thus, radiation damage effect in the LYSO material can be neglected for UFXCT applications up to a certain radiation dose.

### 3.3. Performance at Various Detector Heights and Electron Beam Currents

**Figure 15:**
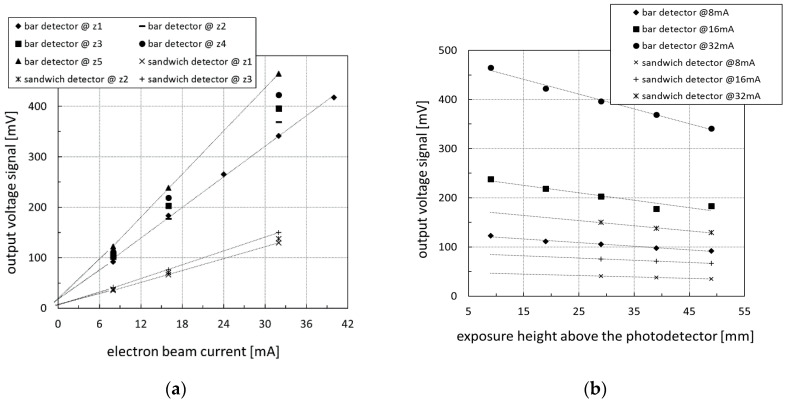
Measured and averaged output voltage signal of LYSO bar and LYSO sandwich multi-plane detectors versus (**a**) various electron beam currents and (**b**) various exposure heights above the photodetector.

In Figure 15, the measured and averaged output voltage signals of both multi-plane detector types is shown for different electron beam currents and at various exposure heights. Figure 15a reveals for both MPDs an excellent voltage output linearity versus the detected X-ray fluxes. Even the subsequently inserted linear trend lines crossed the *y*-axis at the previously determined corresponding voltage offset values. In Figure 15b, there was also very good linearity for different exposure heights that can be discovered. This was important because it clearly confirmed that light losses occurring at the several optical transition zones in the sandwich detector design were negligible. Furthermore, the linear detector signal responses proved that the single multi-plane detector operated identically to commercially available scintillation multi-pixel detectors.

Thus, the more than two times higher scintillation light signal strength obtained from the bar detector was doubtlessly identified as a result of both the parallax effect and the internally generated X-ray scattering (see Figure 13b). In Table 2, calculated signal ratios for the directly exposed bar detector versus sandwich detector are compiled for various electron beam currents and detector exposure heights. As can be seen, there were no trends, and the mean signal ratio value of 2.63 quantified the adjacent LYSO volume height above and below the exposed LYSO bar volume to be 1.63 mm, respectively.

**Figure 16:**
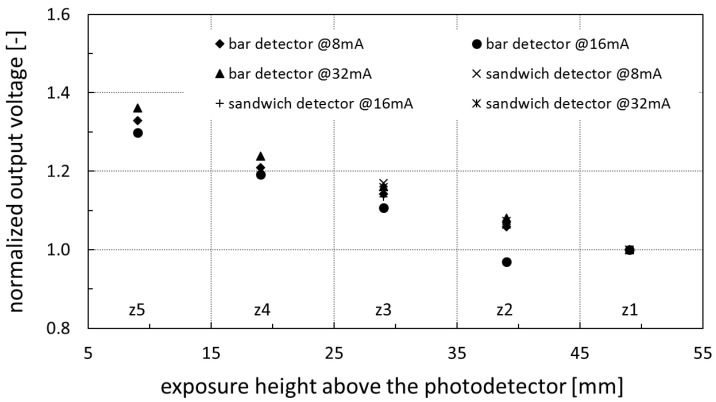
Normalized output voltage versus exposure heights *z*_1_ − *z*_5_ above the photodetector for various electron beam currents and both MPD module configurations.

Consequently, the sandwich design also works properly and without any signal degradation that is, finally, shown in the normalized voltage output plot in Figure 16 that delivers a collective scintillation light output attenuation factor of about −8 m^−1^. The last measurement in the very center of the light-guide material at *z*_6_ = 34 mm, i.e., between two LYSO detection volumes, verified their insensitivity to X-rays.

## 4. Conclusions

In this paper, a novel multi-plane detector (MPD) concept for the application in the multi-plane ultrafast electron beam computed tomography (MP-UFXCT) was presented. The simple but functional MPD is based on an optically clear scintillation material that is coupled to an avalanche photodiode. The scintillation material for each detector channel is extended along with the entire CT scanning height and transfers internally generated scintillation light to the photodetector that is placed outside the scanning range.

For the verification of the multi-plane detector concept, evaluation MPD modules were manufactured and evaluated. As scintillation material cerium doped lutetium yttrium oxyorthosilicate (LYSO:Ce) was chosen. Two different scintillation compositions were tested: A monolithic bar with a volume of 2 × 4 × 50 mm^3^ and a sandwich setup comprising three small LYSO volumes of 2 × 4 × 2 mm^3^ that were connected to light-guiding material of total volume of 2 × 4 × 44 mm^3^. As a photodetector, an adapted avalanche photodiode array (S8550) was used to design a seamlessly stackable detector frontend. Finally, an evaluation electronics was designed for fast detector signal processing up to 1 MHz.

Investigations at different detector heights and electron beam currents revealed excellent linear detector responses and long-term stabilities. Thus, the functionality of such composed multi-plane detectors is identical with commercially available multi-pixel scintillation detectors that are similarly assembled but, respectively, connected to separate electronics. Furthermore, the LYSO sandwich MPD delivered identical signal qualities as the LYSO bar MPD. Thus, not only MP-UFXCT with increased scanning heights becomes possible but also more complex MPD modules.

The next steps are (a) the development of a complete electronically assembled eight-channel multi-plane detector module that can be seamlessly arranged around a transparent target and (b) the design of an analog-to-digital signal converting and detector data-transferring concept that samples the signals of all detector modules with a frequency of *f*_Samp_ = 2 MHz in parallel.

## Figures and Tables

**Table 1:** Selected properties of inorganic scintillation materials useable for multi-plane detector applications [32].

style="border-top:solid thin;border-bottom:solid thin">Scintillation Material	style="border-top:solid thin;border-bottom:solid thin">Light Yield [photons/keV]	style="border-top:solid thin;border-bottom:solid thin">1/e Decay Time [ns]	style="border-top:solid thin;border-bottom:solid thin">Wavelength of Maximum Emission λm [nm]	style="border-top:solid thin;border-bottom:solid thin">Refraction Index at λm	style="border-top:solid thin;border-bottom:solid thin">Density [g/cm^3^]	style="border-top:solid thin;border-bottom:solid thin">Hygro-Scopic
BGO	8–10	300	480	2.15	7.13	no
CsI(TI)	54	1000	550	1.79	4.51	slightly
NaI(TI)	38	250	415	1.85	3.67	yes
LYSO:Ce	32	41	420	1.81	7.1	no
LSO	32	40	435	1.82	7.4	no
YAP	18	27	350	1.94	5.55	no

**Table 2:** Calculated signal ratios for directly exposed bar versus sandwich detector for various electron beam currents and detector exposure heights *z*.

style="border-top:solid thin;border-bottom:solid thin">	style="border-top:solid thin">Signal Ratios of Scintillation Bar Versus Sandwich Detector		
	style="border-bottom:solid thin">8 mA	style="border-bottom:solid thin">16 mA	style="border-bottom:solid thin">32 mA
>***z*_1_ = 49 mm**	>2.63	>2.75	>2.63
>***z*_2_ = 39 mm**	>2.59	>2.50	>2.68
style="border-bottom:solid thin">***z*_3_ = 29 mm**	style="border-bottom:solid thin">2.57	style="border-bottom:solid thin">2.68	style="border-bottom:solid thin">2.64
